# Rabies in a postpandemic world: resilient reservoirs, redoubtable riposte, recurrent roadblocks, and resolute recidivism

**DOI:** 10.1186/s44149-023-00078-8

**Published:** 2023-05-19

**Authors:** Charles E. Rupprecht, Philip P. Mshelbwala, R. Guy Reeves, Ivan V. Kuzmin

**Affiliations:** 1 College of Forestry, Wildlife & Environment, College of Veterinary Medicine, Auburn University, Auburn, AL 36849 USA; 2 School of Veterinary Science, University of Queensland, Gatton, Australia; 3 Department of Veterinary Medicine, Faculty of Veterinary Medicine, University of Abuja, Abuja, Nigeria; 4 Max Planck Institut Für Evolutionsbiologie, 24306 Plön, Germany; 5 Department of Pathology, University of Texas Medical Branch, Galveston, TX 77555 USA

**Keywords:** Diagnosis, Epidemiology, *Lyssavirus*, Neglected Tropical Diseases, Pathogenesis, Prophylaxis, Rabies, Surveillance, Vaccination, Zoonosis

## Abstract

Rabies is an ancient disease. Two centuries since Pasteur, fundamental progress occurred in virology, vaccinology, and diagnostics—and an understanding of pathobiology and epizootiology of rabies in testament to One Health—before common terminological coinage. Prevention, control, selective elimination, and even the unthinkable—occasional treatment—of this zoonosis dawned by the twenty-first century. However, in contrast to smallpox and rinderpest, eradication is a wishful misnomer applied to rabies, particularly post-COVID-19 pandemic. Reasons are minion. Polyhostality encompasses bats and mesocarnivores, but other mammals represent a diverse spectrum of potential hosts. While rabies virus is the classical member of the genus, other species of lyssaviruses also cause the disease. Some reservoirs remain cryptic. Although global, this viral encephalitis is untreatable and often ignored. As with other neglected diseases, laboratory-based surveillance falls short of the notifiable ideal, especially in lower- and middle-income countries. Calculation of actual burden defaults to a flux within broad health economic models. Competing priorities, lack of defined, long-term international donors, and shrinking local champions challenge human prophylaxis and mass dog vaccination toward targets of 2030 for even canine rabies impacts. For prevention, all licensed vaccines are delivered to the individual, whether parenteral or oral–essentially ‘one and done’. Exploiting mammalian social behaviors, future ‘spreadable vaccines’ might increase the proportion of immunized hosts per unit effort. However, the release of replication-competent, genetically modified organisms selectively engineered to spread intentionally throughout a population raises significant biological, ethical, and regulatory issues in need of broader, transdisciplinary discourse. How this rather curious idea will evolve toward actual unconventional prevention, control, or elimination in the near term remains debatable. In the interim, more precise terminology and realistic expectations serve as the norm for diverse, collective constituents to maintain progress in the field.

## Introduction

Rabies is a quintessential disease of nature, predating the domestication of *Canis* spp., but is historically associated with dogs in the minds of humankind (Rupprecht et al. 2020). Technical advances throughout the twentieth century provided pivotal insights into the etiology, diagnosis, pathobiology, prevention, control and potential treatment of this acute, progressive encephalitis (Tarantola 2017). Such progress tempted multiple authors to apply the term ‘eradication’ in relation to rabies (Brochier et al. 1991; Flamand et al. 1992; Terré et al. 1996; Grimm 2002; Larghi 2004; Kasempimolporn et al. 2008; Pastoret et al. 2014; Thomson and Penrith 2017; Sartore et al. 2018; Reece 2019; Mubashir and Hussain 2021; Lojkić et al. 2021; Aréchiga Ceballos et al. 2022; Shafaati et al. 2023). Whether due to linguistic license or singular optimism, to muse upon the fantastic and apply such terminology to this zoonosis globally seems in conflict with prior infectious disease concepts, related in no small part to limitations in ‘…feasibility, infrastructure, funding and political will…’ (Dowdle and Cochi 2011). In retrospect, the COVID-19 pandemic chastised our collective epidemiological tomfoolery and blunted global confidence over basic conventional prevention and control (Warrell 2021; Gongal et al. 2022; Nadal et al. 2022a, b; Tidman et al. 2022; Goel et al. 2023).

Not unexpectedly, neglected tropical diseases (NTDs) were impacted disproportionately by the COVID-19 pandemic. Rabies was no exception. For example, in the USA, an uptick of five human deaths occurred (including unprecedented human prophylaxis failure), and dog importation was restricted (Pieracci et al. 2021; Ma et al. 2023). In South America, Peru grappled once again with canine rabies (Raynor et al. 2021). Despite preliminary progress, basic animal control requires a major reset in Haiti (Kunkel et al. 2021). In Sri Lanka, where a prepandemic prevention and control program made significant headway, elimination no longer appears imminent (Kanda et al. 2021). Indirectly, the enactment of COVID-19-associated global lockdowns might have temporarily reduced the incidence of dog bites, but supply chain interruptions also affected the availability of biologics and quality of medical care, including the threat of counterfeit vaccines (Henson et al. 2020; Saleem et al. 2021; Siddiqui et al. 2021). The broader controversy on human vaccine hesitancy extended to pet owners with reluctance to receive rabies vaccines (Kogan and Rishniw 2022). Rabies reports rose throughout sub-Saharan Africa (Goel et al. 2023). On the subcontinent, the burden of rabies in India was unsurpassed by any other country, even before the pandemic. Within the Indian state of Kerala, nearly 2 million dog-bite incidents were recorded during 2022, including rabies occurrence despite vaccination (Thiagarajan 2022). The death of at least six persons who began rabies prophylaxis triggered public anxiety and a major investigation into the relative quality of available biologics (Bajeli-Datt 2022).

Major repercussions from the global pandemic continue to ripple throughout the rabies profession, raising significant issues. Irrespective of these relatively recent events, why is rabies *not* a candidate for eradication? What is the true status of rabies prevention in the near term? Emerging technologies should bolster conventional disease control efforts, but will new biologics or concepts under development recoup contemporary setbacks and jumpstart elimination? The objectives of this communication are to present a brief update on current global efforts on rabies and its management based upon recent contributions to the literature and to provide a prospective rationale as to the realistic limitations of these actions within the larger historical context of the field.

## Viral systematics and ramifications of an evolving taxonomy

**Fig. 1:**
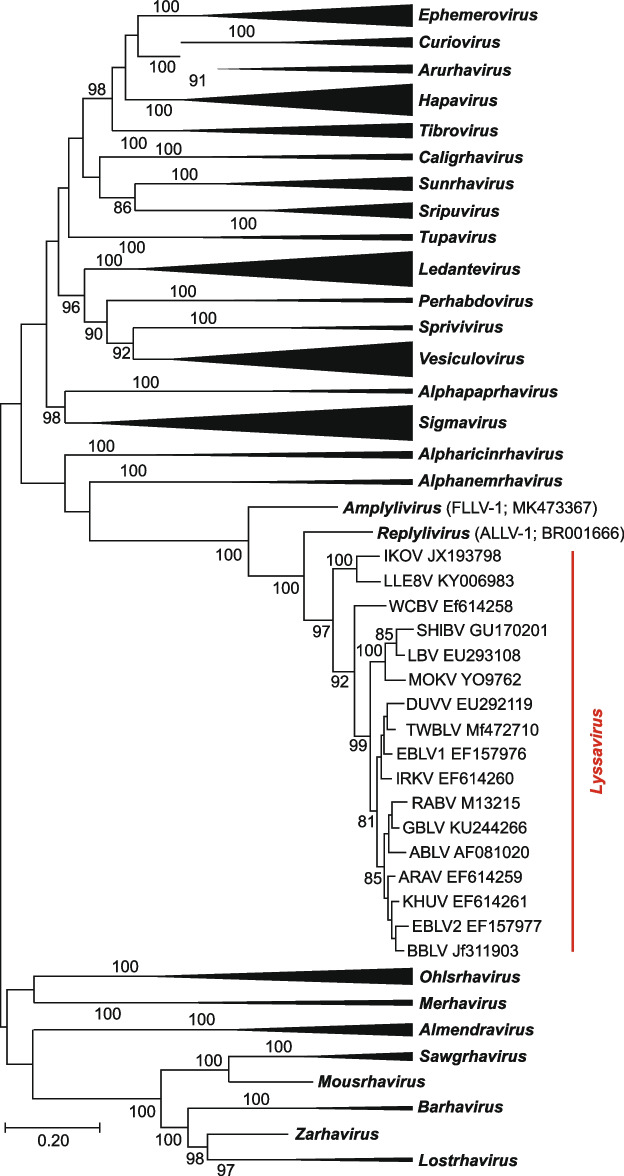
Phylogenetic placement of lyssaviruses within the subfamily *Alpharhabdovirinae*, family *Rhabdoviridae*. The evolutionary history was inferred from an alignment of the complete L protein sequences of animal rhabdoviruses, using the Maximum Likelihood method. Initial tree(s) for the heuristic search were obtained automatically by applying Neighbor-Joining and BioNJ algorithms to a matrix of pairwise distances estimated using a JTT model. Statistical significance was assessed via 100 bootstrap replicates (values > 70 are shown for key nodes)

Reflections on disease prevention, control, elimination, and eradication benefit from introspection on how various pathogens arose, emerged and perpetuated. However, despite the historical record of rabies and its widespread occurrence in humans, domestic animals and wild mammals, lyssavirus origins and evolution remain puzzling. Genetic distances within the *Lyssavirus* genus (Fig. 1) are much shorter than those in other rhabdovirus genera (Walker et al. 2018). Some investigators have suggested that such genetic homogeneity may reflect a relatively younger age of lyssaviruses (Bourhy et al. 2005).

In addition, for decades, the *Lyssavirus* genus was an “orphan” within the larger rhabdovirus phylogeny, without any major clues on progenitors. Only in 2019 was a lyssavirus-related rhabdovirus identified in a tree frog, *Dryophytes cinereus*, and another lyssavirus-related virus was detected in a lizard, *Anolis allogus* (Horie et al. 2021). These viruses were not monophyletic to each other (Fig. 1). Therefore, they were classified into two separate genera, with one species each, *Replylivirus* and *Amplylivirus,* by the International Committee on Taxonomy of Viruses (ICTV). Indeed, the question remains, did lyssaviruses originate from amphibian or reptilian rhabdoviruses (which sounds rather unlikely from an ecological perspective, given that the majority of lyssaviruses circulate in bats)?

Approximately 65 million years ago, at the end-Cretaceous mass extinction, most mammals were small, nocturnal, and insectivorous before their substantial ecological release and ensuing adaptive radiation of body sizes and niche occupations from presumed lessened interspecific competition (Grossnickle and Newham 2016). Perhaps an insect-borne lyssa-like virus (yet to be discovered) was a progenitor for related viruses infecting terrestrial insectivorous animals, such as frogs, lizards, and later, mammals, including bats (Shope 1982). In any scenario, the remarkable genetic similarity between the replylivirus, amplylivirus, and lyssaviruses points to an ancient origin of all these viruses. Biogeographically, both the amplylivirus and replylivirus were detected in the New World (North America and Cuba, respectively), whereas the greatest lyssavirus diversity is seen in the Old World. Africa (Nel and Rupprecht 2007) or Eurasia (Hayman et al. 2016) were suggested as the most likely areas of *lyssavirus* origination. However, we still do not have an instrument for retrospective estimation of age and evolutionary traits of viral genomes subjected to strong purifying selection, especially when only a limited set of recent samples is available (Wertheim and Kosakovsky Pond 2011; Wertheim et al. 2013).

When did rabies arise? Most molecular clock estimates for lyssaviruses were performed on sequences collected during recent decades. Evolutionary rates in a set of sequences are time dependent, increasing toward the present, because the transient mutations have yet to be removed by purifying selection. When the rate of decay dynamics is considered, ‘viral age’ increases by several orders of magnitude (Ho et al. 2011; Membrebe et al. 2019). We consider the discovery of lyssa-like rhabdoviruses in amphibians and reptiles as a confirmation of opinions expressed earlier that lyssaviruses are not recent over thousands of years but rather evolved over tens of millions of years (Shope 1982; Rupprecht et al. 2017). Lyssaviruses may have descended and disseminated from ancient Gondwana via now extinct mammals and later proto-bats in the Eocene. Given the susceptibility of birds and the presence of amphibian and reptile lyssa-like viruses confined thus far to the New World, such ancestral viruses may have already been present broadly by the time of ongoing continental drift during the Late Cretaceous, while mammals were in transition.

**Fig. 2:**
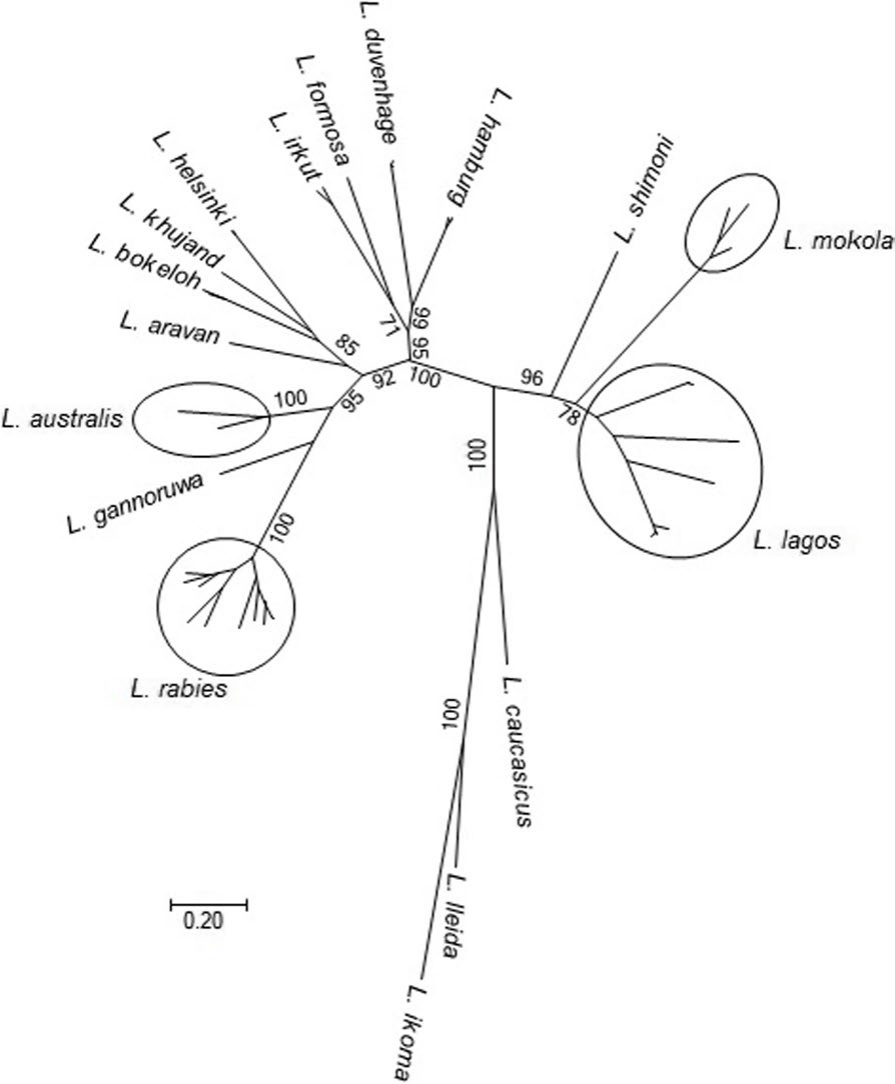
Phylogenetic structure of the *Lyssavirus* genus based on the complete N gene sequences of representatives from each currently recognized species. The topology was inferred using the Maximum Likelihood method. Initial trees for the heuristic search were obtained automatically by applying Neighbor-Joining and BioNJ algorithms to a matrix of pairwise distances estimated using a JTT model. Statistical significance was assessed via 100 bootstrap replicates (values > 70 are shown for key nodes)

Although relative ecological compartmentalization was realized, rabies was thought to be caused by a single entity until the detection of ‘rabies-related’ viruses during the 1950s (Shope 1982). At present, ICTV recognizes 17 *lyssavirus* species (Fig. 2). Most of these viral species are associated with bats. The only “exception” from that rule was suggested to be rabies virus (from a taxonomic perspective, the virus lineages included in the species *Lyssavirus rabies*), which circulates among bats, mesocarnivores, potentially in New World nonhuman primates, such as marmosets, and perhaps even temporarily in certain ungulates under ideal ecological conditions, such as kudu antelopes (Marston et al. 2018; Ness et al. 2023). Such an assessment may be correct only as long as we consider that the assignment of lyssaviruses to the host species is correct.

In general, taxonomy is only a tool that humans use to break the continuity of living forms in an attempt to classify them by specific characteristics. This demarcation is neither absolute nor ideal and may vary depending on the set of criteria used. The present demarcation of lyssaviruses includes genetic sequence identity, phylogenetic tree topology, serologic cross-reactivity, and ecological traits, such as host range, geographic distribution, and pathobiology (Rhabdoviridae. Virus Taxonomy: 2021 Release. EC 53, Online, July 2021). Not all of these criteria are met for every lyssavirus species identified to date (Fig. 2). For example, “Phylogroup I” lyssaviruses cross-neutralize each other, as do “Phylogroup II” lyssaviruses (Badrane et al. 2001; Hanlon et al. 2005; Horton et al. 2010). Genetic distances between representatives of *Lyssavirus gannoruwa* and *L. australis*, as well as between related Eurasian bat lyssaviruses such as *L. helsinki*, *L. bokeloh*, and *L. khujand*, are very close to the distances within *L*. *rabies* (80–81% nucleotide identity for the nucleoprotein gene). In contrast, genetic distances between lineages of *L. lagos* (approximately 79% nucleotide identity for the nucleoprotein gene) are longer than the intraspecies demarcation limits set by ICTV (Markotter et al. 2008).

Taxonomic changes may be cogent in both extremes. For example, all “Phylogroup I” lyssaviruses may be considered members of one species (e.g., *L. rabies*), but the same would be hard to reconcile with “Phylogroup II” lyssaviruses, given that *L. lagos* and *L. mokola* are certainly different viruses by various characteristics, and *L. shimoni*, in some phylogenetic constructions, is monophyletic to *L. lagos* but in others is ancestral to both *L. lagos* and *L. mokola* (Kuzmin et al. 2010).

As an alternative extreme, one may consider that some of the *L. rabies* lineages that demonstrate strict association with a specific host and geographic area are separate lyssavirus species, at least from ecological perspectives. Indeed, even for *L. rabies* lineages, most cross-species transmission events from the reservoir usually lead to dead-end infections or only short-term outbreaks (Streicker et al. 2010; Kuzmin et al. 2012). The present taxonomy balances such extremes, trying to accommodate the multidimensional demarcation of lyssavirus species. However, what one is facing now is that the ICTV is attempting to classify multiple viral genomes identified during metagenomic studies, rather than entire viruses. In such settings, neither serologic, pathobiologic, nor ecologic properties of viruses are taken into account. With the entire taxonomy moving toward “genotyping”, genetic criteria are going to become overarching. As such, the present structure of the *Lyssavirus* genus will probably be reassessed in the near future.

Another empirical caveat associated with modern viral taxonomy is the frequent changing of species names. This alteration occurred several times over recent years, along with attempts to establish a binomial system. This binomial system, although resembling that of “larger” biology, is not exactly Linnaean but operates with “free-form” species epithets (Zerbini et al. 2022).

Species epithets are not uniform. They are used either in English, Latin (sometimes in improper form), or via historical terms. Considering the long iterative process of virus species renaming, we may expect that it will continue. Importantly, however, the concept of ‘viruses’ (biologic entities) is not the same as ‘virus species’ (taxonomic entities). Viral names are not a subject of ICTV policies and remain unchanged (and can be abbreviated, unlike species names). For example, rabies virus (RABV) is still rabies virus, whereas the species name was changed to *Rabies lyssavirus* and then to *Lyssavirus rabies*. Similarly, European bat lyssavirus, type 1 (EBLV-1) is still the same biologically, even though the species name was changed to *European bat 1 lyssavirus* and then to *Lyssavirus hamburg* (https://pubmed.ncbi.nlm.nih.gov/36437428/).

Regardless of the varying complexities of taxonomic ruminations, new lyssaviruses await detection and characterization and are particularly relevant regarding global disease emergence and public health implications (Fooks et al. 2021). To date, there are only a handful of primary human deaths attributed to ‘rabies-related’ lyssaviruses in Africa, Australia and Eurasia (van Thiel et al. 2009; Markotter and Coertse 2018; Merritt et al. 2018; Poleshchuk et al. 2023). No documented human postexposure prophylaxis (PEP) failures are yet known due to disparate lyssaviruses, despite the lack of veterinary vaccine breadth and occasional breakthrough infections from case reports in domestic animals (Coertse et al. 2021). Often, such incidents are viewed as biological curiosities, divorced from the reality that most human rabies cases are never confirmed by laboratory testing. A recent note of the identification of West Caucasian Bat Virus from a cat in Italy, > 2,000 km and 18 years from the first isolation in the Caucasus mountains, should provide some pause for concern over surveillance limitations and biosecurity, as well as the objectivity of a truly ‘rabies-free’ Europe (Leopardi et al. 2021; Vega et al. 2021). Irrespective of the existence of bat lyssaviruses, rabies in mesocarnivores perpetuates in the eastern portion of the continent and may become more exacerbated given ongoing strife.

At a minimum, taxonomic recognition of antigenically distinct lyssaviruses beyond RABV, without enhanced laboratory-based surveillance and safe and cross-reactive biologics, challenges any notion of simplistic rabies eradication. Conversely, viral taxonomy may be used by some for fleeting alterations in operational definitions (*e.g.*, that rabies is caused by RABV alone, that wildlife rabies is comparatively unimportant, or that non-RABV causes only a ‘rabies-like’ disease) and could provide more palatable, short-term public health solutions to seemingly bend nature to the ephemeral biopolitical will.

## Polyhostality: resilient wildlife and dogs

Considering the taxonomic breadth of extant warm-blooded vertebrates, more than 16,000 potential species are considered susceptible to *lyssavirus* infection (Paarmann 1955; Baby et al. 2015; Gilbert 2018; Rohde and Rupprecht 2020). This wide span reflects in part both viral plasticity and various host conserved factors over evolutionary time, including structural and biochemical features of receptors, replication dynamics, excretion mechanisms, and host innate/adaptive immunity (Conselheiro et al. 2022; Gérard et al. 2022; Lian et al. 2022). The capability of infection for all hosts is dissimilar from perpetuation, as birds have been toothless for millions of years, removing one major anatomical assist for viral entry, notwithstanding the potential capabilities of beaks to create lesions from modern avianized dinosaurs (Rohde and Rupprecht 2020). Any resulting productive infection is impacted at the outset by a time-space continuum overlap, requiring simultaneous engagement of host and pathogen (Jacquot et al. 2022). For example, on certain islands, such as Hawaii, susceptible hosts abound, but splendid isolation in this case of a distant, insular environment is devoid of a pathogen source, unless introduced (Sasaki et al. 1992). Greater proximity to a source also complicates the probability of relative sustainability (Morgan et al. 2020). In addition, such appreciation also implies a relevant, decentralized laboratory-based surveillance system, with more limited bias for the inclusion of wildlife (Wallace et al. 2014). For example, without considering nondomestic reservoirs, Taiwan would not have detected naturally infected bats and ferret badgers (Hsu et al. 2019; Zhao et al. 2019).

**Fig. 3:**
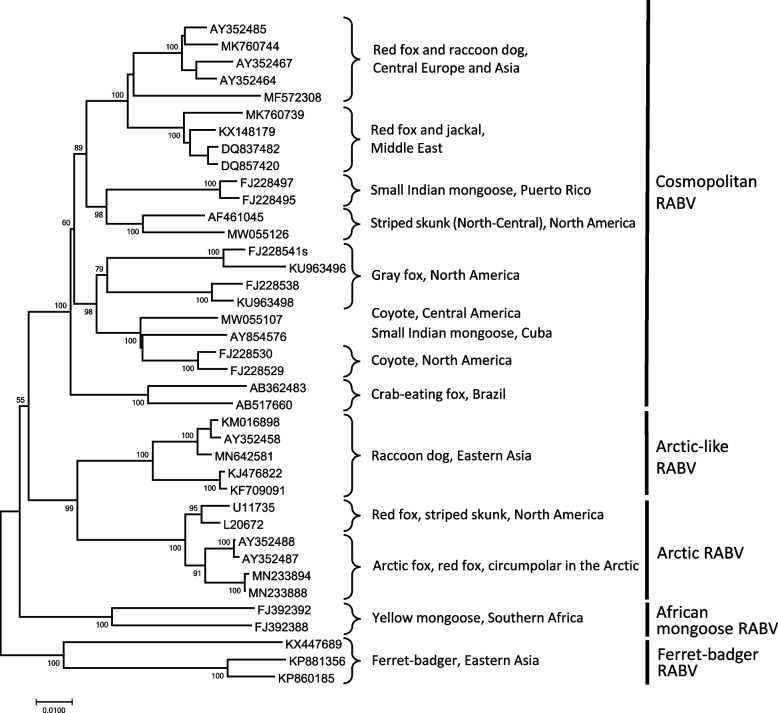
Phylogenetic lineages of rabies viruses (RABV) circulating in wild carnivores that are related to canine rabies viruses. The tree was generated from full-length nucleoprotein (N) gene sequences by the neighbor-joining method supported by 1,000 bootstrap replicates. GenBank accession numbers are shown at tree tips, host species and geographic region are indicated after brackets, and conventional names for RABV lineages are shown on the right

Wildlife rabies began long before the origin of domestic species, with lyssaviruses in bats evolving prior to carnivores (Badrane and Tordo 2001). Among mammalian hosts, the recorded host gamut spans from anteaters to zebras (Obonyo et al. 2014; Grome et al. 2022). Although no marsupials have been reported from Australia yet, even relatively resistant nonplacental taxa, such as New World opossums, are known (Diana et al. 2015). Most ‘spillover’ cases, such as among human victims, are considered dead-end infections unless tissues and organs are used inadvertently for transplantation (Mrzljak et al. 2020). Otherwise, when applied to rabies, intraspecific perpetuation is predictable from mammalian sociobiology, particularly among canids. Arctic foxes are key drivers of infection in circumpolar environments (Ørpetveit et al. 2022). Other fox species serve this role in temperate and tropical environments (Caraballo et al. 2021; Ma et al. 2023; Matulis et al. 2022). Coyotes and jackals are ecological and epizootiological equivalents within both the New and Old Worlds (Shwiff et al. 2008; Ngoepe et al. 2022). Raccoon dogs acquired dog and fox RABV throughout Eurasia (Yang et al. 2017; Shulpin et al. 2018). Direct cross-species transmission (CST) is also inevitable among noncanid carnivores, such as ferret badgers and mongooses. due to a suite of ecological imperatives, such as predator–prey relationships and interspecific antagonistic interactions (Fig. 3). The applicable impacts of this polyhostality suggest that oral vaccination of wildlife may be necessary within the dual context of long-term canine rabies management, as exemplified at the Mexico-USA border and elsewhere (Sidwa et al. 2005; Malan et al. 2022; Ma et al. 2023).

**Fig. 4:**
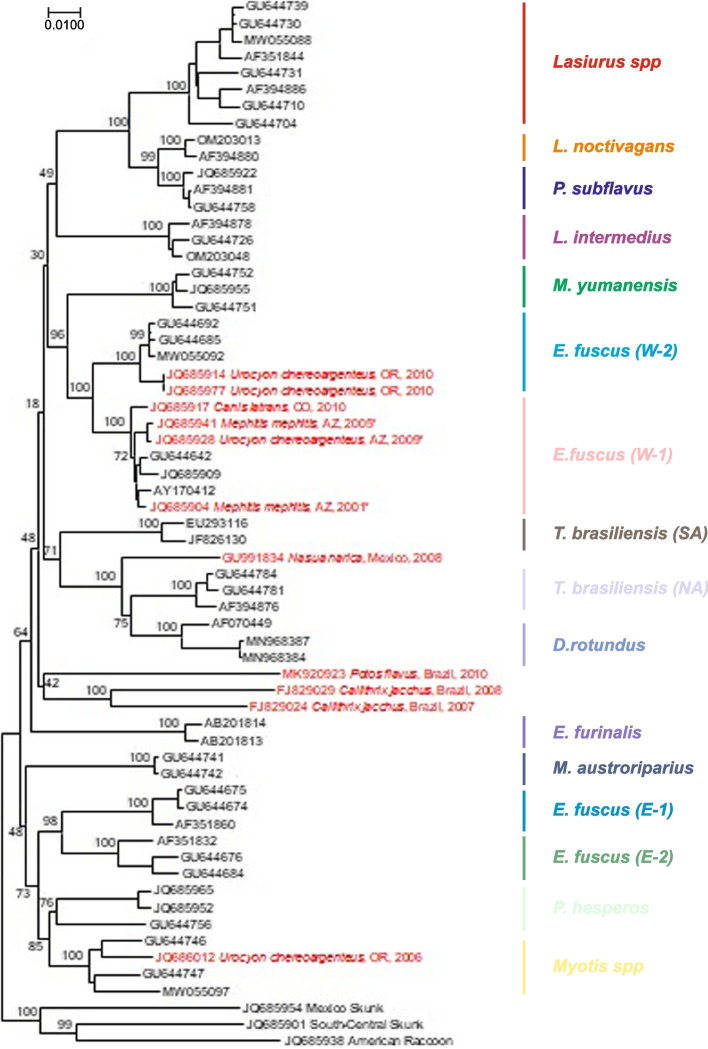
New World bat rabies virus (RABV) diversity (black) in association with infections in carnivores (red). Asterisks indicate viruses recovered during known outbreaks with sustained cross-species transmission of bat RABV among carnivores. Bat species and genera that serve as lineage hosts are indicated on the right. The neighbor-joining tree is based on RABV nucleoprotein (N) gene sequences with 1000 bootstrap replicates (shown as % for key nodes) and rooted with Mexican skunk, South-Central skunk, and raccoon RABV lineages (also related to American bat viruses within the "Indigenous American" RABV lineage)

Similarly, bat to nonbat CST is evident among carnivores and other species, independent of canine lineages (Fig. 4). Compared to domestic dog-wild carnivore CST events, the frequency and mechanisms of host shifts for longer-term perpetuation from bats to other mammals is poorly understood (Kuzmin et al. 2012). Such realities not only demonstrate that rabies will perpetuate even if conventional canine rabies is controlled but also provide evidence of maintenance among carnivores with the opportunity to return from the wild to the domestic animal cycle. Moreover, the complexities of such overt polyhostality not only complicate current plans for prevention, control, and selective elimination but also nullify the suggestions of eradication of this disease, even if RABV remains the only significant *lyssavirus* responsible for CST, disease emergence, and perpetuation (Marston et al. 2017, 2018).

## Resolute burden estimates

How many people die of rabies? No one knows. Why? In part, explanations are similar for the lack of laboratory-based surveillance related to NTD in general (Ghai and Hemachudha 2022). Others are more pathogen specific (Table 1). As a disease related to poverty, individuals, such as children, may not fundamentally understand how rabies is acquired or may not appreciate its severity (Laorujisawat et al. 2022; Mapatse et al. 2022; Zucca et al. 2021). Even if a bite is reflected as a risk factor for disease acquisition, less serious or nonbite exposures may be ignored or not be recognized, such as related to bats, even in highly developed countries (Holzbauer et al. 2023; Ma et al. 2023). Recall bias could also be an issue if exposure occurred months to years previously (Amoako et al. 2021). Considering the lack of therapy, rather than being refused care or even locked away and ignored in a medical ward as a pariah, families may prefer patients to die at home with a modicum of empathy and simple palliation (Warrell et al. 2017). Although rabies has been described clinically and characterized by its often unique manifestations, such as hydrophobia, practitioners may not appreciate more subtle or atypical signs, such as in the paralytic form (Hemachudha and Hemachudha 2021). Similarly, the disease may be masked by acute trauma or misdiagnosed as related to other forms of encephalitis (Mallewa et al. 2007; Mudiyanselage et al. 2016; Bhat et al. 2021). Despite a number of highly sensitive and specific diagnostic tests recognized by WOAH and WHO, there may be no suitable laboratory facility in the locality where human deaths occur, such as in rural areas (Rana et al. 2020). Families traumatized by the sudden death of a family member may not consent to invasive procedures postmortem. Certain religious practices, such as requiring relatively rapid internment, may preclude desired sample collection or accurate clinical case reporting.

**Table 1:** Factors related to an underreporting of rabies cases

Number	Factors
1	Ignorance of causality
2	Nonrecognition of exposure
3	Longer incubation periods
4	Futility of treatment
5	Atypical clinical signs
6	Burial practices
7	Lack of local testing facilities
8	Fear of contamination
9	Misdiagnosis
10	Decreasing autopsies
11	Nonnotifiability
12	Self-perpetuating cycle of neglect

A few inferences of the extent of global human rabies appear, which of course are not inviolated. Epidemiologically, values should vary spatiotemporally. For example, the annual number of human rabies deaths in Africa and Asia was estimated at between 24,000 and 93,000 cases and to cost more than $583 million (Knobel et al. 2005). Another often quoted global model calculated 25,000 to 159,000 human deaths, over 3.7 million disability-adjusted life years, and $8.6 billion economic losses annually (Hampson et al. 2015). More recently, an investigation extrapolating from a 2019 Global Burden of Disease Study suggested that overall fatalities declined over a decade, with a range of 6,019 to 17,939 human deaths (Gan et al. 2023). Clearly, such data are important for appropriate planning, to generate resources, to measure success, etc., but quite difficult to generate (Taylor et al. 2017; Minhaj et al. 2023).

## Progress and attempted quick returns in the face of recurrent setbacks in the global elimination of human rabies by dogs

**Fig. 5:**
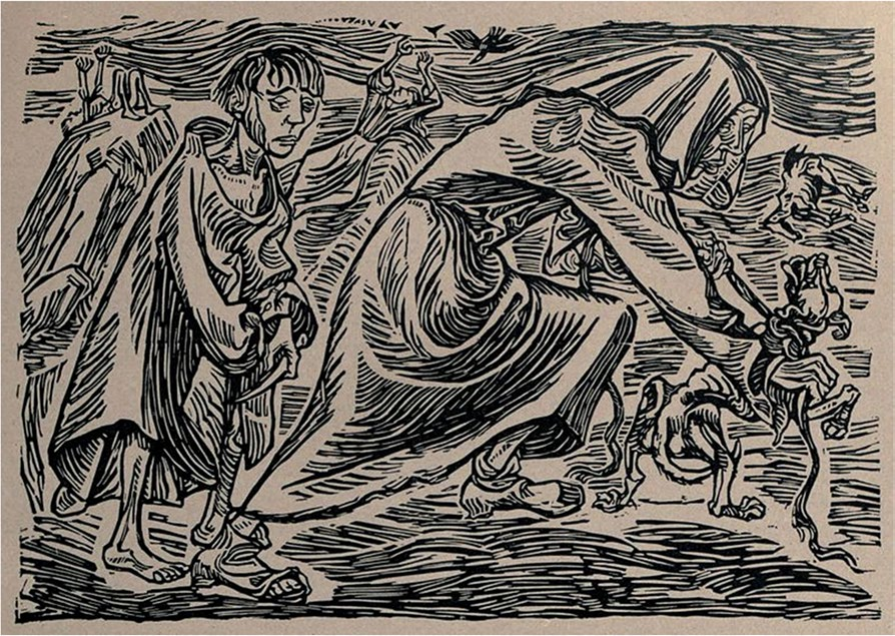
Hundefängerin (dog chasers), Ernst Barlach, woodcut, ca. ~ 1919. From: Die Kunst. Monatshefte für freie und angewandte Kunst, vol. 4 https://commons.wikimedia.org/wiki/File:Hundef%C3%A4nger,_Ernst_Barlach.jpg3, 1921, p143

**Fig. 6:**
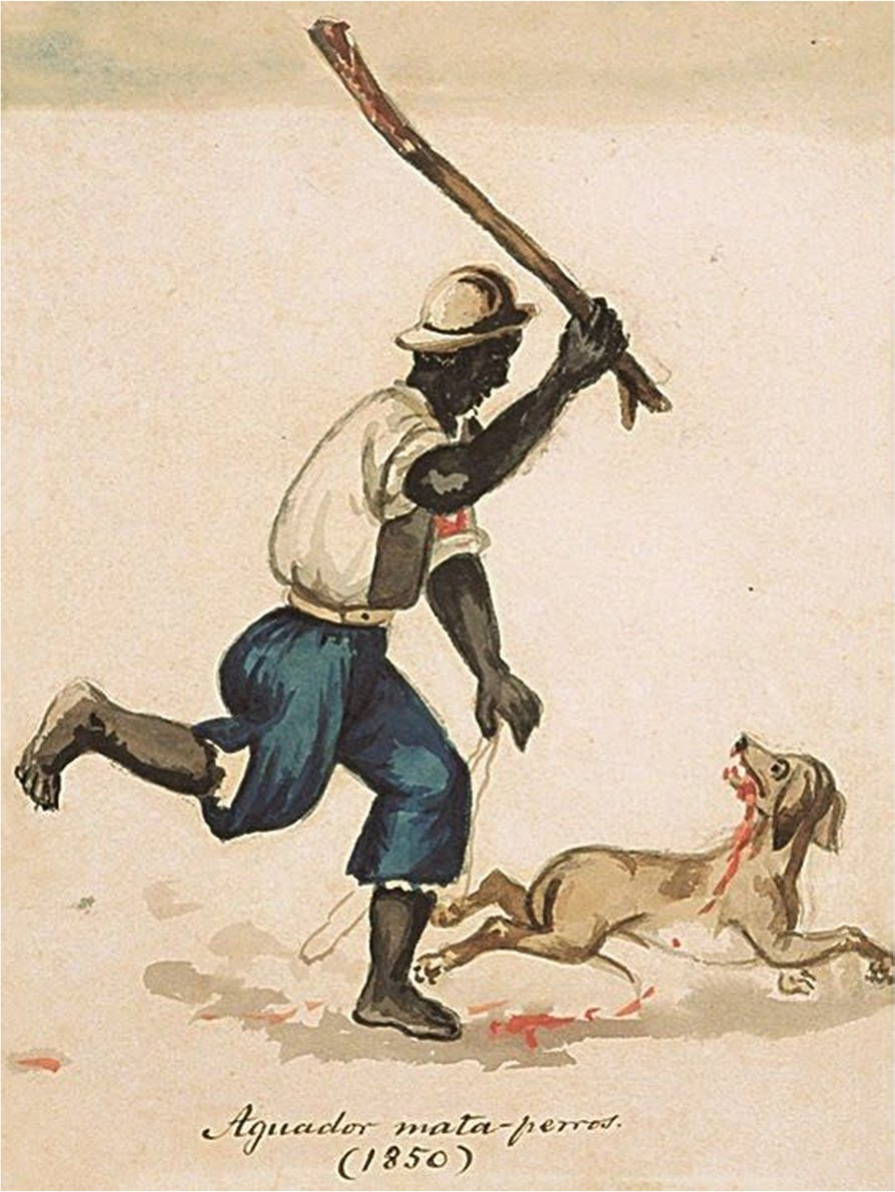
‘Aguador mata-perros ‘ (waterseller dog-killer), watercolor painting by Francisco ‘Pancho’ Fierro Palas (Lima, 1850). https://commons.wikimedia.org/wiki/File:Aguador_mata-perros_(1850).jpg

For thousands of years to the present, most human rabies fatalities occurred after dog bites. Hence, the current focus on the prevention, control, and selective elimination of canine rabies is an integrated reality built upon centuries of prior experience, unfortunately often driven by fear and indiscriminate culling of the pariah dog, as represented by the effigy and art of everyday life (Figs. 5 and 6). Large parts of Europe accomplished this task long before the advent of vaccines, largely by population reduction, leashing, and use of muzzles. Other locales, usually insular, apparently never reported canine rabies or had an abbreviated interlude, relying upon geographic isolation and quarantine. For example, in addition to an early introduction event during 1866-67, canine rabies did not become established in Australia, and an active program to prevent its reoccurrence prevailed (Sparkes et al. 2015).

Building upon Pasteur’s experimental work, mass dog vaccination (MDV) began during the 1920s in Japan (Umeno and Doi 1921). Rabies was eliminated there by 1957 through coordinated canine vaccination and dog management (Kamata et al. 2023). Elsewhere, twentieth century progress occurred throughout the Americas after the original introduction of canine rabies via European colonization. In addition to culling, leash laws, *etc.*, Canada and the USA implemented dog vaccination following World War II (Rosatte 1988; Ma et al. 2023). Both countries successfully controlled canine-mediated rabies with a regional public health strategy. Licensing of owned dogs supported animal control activities, and the outright killing of free-ranging dogs moved gradually toward a policy for their capture and holding at ‘pounds’ for adoption or euthanasia (Fig. 6). Comparatively, by the end of the twentieth century, few human cases appeared after elimination of canine rabies, acquired through imported human cases (exposed to dogs abroad) or after indigenous wildlife exposures (Daniels et al. 2020; Kunkel et al. 2022; Ma et al. 2023).

After 1993, Mexico's national program implemented massive MDV, eliminating canine-mediated human rabies deaths (González-Roldán et al. 2021). Estimates suggest that the program prevented approximately 13,000 human rabies deaths by investing more than $300 million in surveillance, prevention, and control. Mexico received validation from the WHO as a country free of canine-mediated human rabies deaths, in line with the regional program for elimination (Del Rio Vilas et al. 2017). However, because of enzootic wildlife rabies, even after curtailment of canine rabies, the challenges of human prevention remain, especially within remote communities. For example, recently, a seven-year-old boy died after being bitten by a bat in an impoverished rural community of Oaxaca (News Desk 2022). The child only reported to the hospital 20 days after exposure, already exhibiting symptoms consistent with rabies. This exposure occurred 100 km from the main city, with no rabies vaccines. Another ill child was also suspected to be infected and died. Authorities coordinated a public health response comprised of renewed educational outreach and local animal vaccination. While canine rabies elimination alleviates a significant burden, such success does not erase the public health and veterinary responsibilities associated with the disease and its ongoing prevention due to infected wildlife.

In addition to Mexico, multiple countries within Latin America (e.g., Argentina, Chile, etc.) achieved significant progress toward eliminating canine-mediated human rabies deaths through MDV programs, which began via coordinated efforts with PAHO during 1983, first targeting major urban centers in excess of 100,000 human inhabitants (Freire de Carvalho et al. 2018). Many countries within the Americas (except Bolivia, Haiti, etc.) are considered canine rabies-free, including the Caribbean region. Whether most countries will participate in a similar process of validation as accomplished by Mexico remains speculative. Thus, over the past 200 years, multiple localities have attained freedom from canine rabies and prevented its re-establishment, including western Europe, most of the Americas and the Caribbean, Japan, Australia and Pacific Oceania, even though other lyssaviruses or RABV variants of wild mesocarnivores and bats perpetuate (Rupprecht et al. 2018). Today, the major burden lies within the affected tropical portions of Asia and Africa (Rupprecht et al. 2022).

Recognizing progress in the Americas and the major needs within the Old World, in 2010, with support from the Bill and Melinda Gates Foundation (BMGF), proof-of-concept canine rabies elimination projects were initiated at sites in the Philippines, South Africa and Tanzania (Elser et al. 2018). These projects demonstrated the feasibility of local prevention and control in support of broader disease elimination plans (Shwiff et al. 2016; Hatch et al. 2017; Miranda et al. 2017; Mpolya et al. 2017; LeRoux et al. 2018). In addition, the building blocks erected from these projects via BMGF support continued to bear weight. For example, in Tanzania, on 14 February 2022, a 12 year-old village boy was bitten by an unfamiliar dog in the Ngorongoro Conservation Area. Weeks later, he died within two hours of presentation at a hospital in Karatu after displaying signs on 6 March, consistent with rabies (i.e., known locally as kichaa cha umbwa, Swahili for “madness of dogs”). Although this child was not saved, quick-acting hospital staff, after learning of other exposed children in the same village, notified public health authorities, such that 10 other at-risk persons were able to be vaccinated, exemplifying rapid risk communications and integrated preventative actions by a diverse biomedical team (Resolve To Save Lives (RTSL), 2022). Such an effective local response epitomizes a larger national plan for rabies management (Tanzania 2019). Hence, while the country is still enzootic for canine rabies, long-standing public – private partnerships and initiated local efforts continue to make major inroads toward prevention and control (Lushasi et al. 2020).

In the KwaZulu-Natal (KZN) province of South Africa, the immediate benefits of the BMGF project were appreciable in limiting human deaths. However, progress appeared rather short-lived and not readily extrapolated to the rest of the country. Overall, national human rabies cases reported from 2008 to 2018 remained largely unchanged (Weyer et al. 2020). Within KZN province, a cross-sectional observational investigation showed that less than 70% of dogs were vaccinated. Potential factors for less-than-ideal canine vaccination coverage included animal age, population turnover, the number of free roaming dogs, and husbandry practices in certain high-risk areas (Hergert et al. 2016). A qualitative study in the province during 2017-18 found a lack of broad public knowledge on rabies, economic constraints for vaccination, and the unavailability of PEP at local health facilities (Hadebe and Sibiya 2020). This was especially alarming, given the zero human deaths recorded in KZN during 2014 from the BMGF supported project (LeRoux et al. 2018). In addition, although dogs are the primary RABV reservoir in South Africa, jackals are also associated with the disease (Mogano et al. 2022; Ngoepe et al. 2022). With the maintenance of canine rabies and in the absence of broader MDV, the impacts for proper health care delivery and PEP after animal exposures remain substantial (Whitbread et al. 2022). Recently, in the popular tourist area of Nelson Mandela Bay Municipality, an unprecedented rabies outbreak began. Between 1st January 2021 and 13th February 2022, 436 rabid dogs and five human fatalities were reported during a concomitant outbreak of foot and mouth disease (Hill 2022; Ravensberg et al. 2022).

In the third BMGF-supported project, in the Philippines, from 2010 to 2017, the number of rabies-free provinces and municipalities increased from three to 49 (Leonardo et al. 2020). The overall progress was associated with greater veterinary services involvement, broad public support, communal advocacy, increased health communications, focused community education, and One Health linkages (Medina et al. 2016; Valenzuela et al. 2017; Barroga et al. 2018; Deray et al. 2018; Amparo et al. 2019). However, in the Metro Manila area, between 2006 and 2015, there was no decline in deaths or in the catchment area from which human rabies cases occurred (Guzman et al. 2022). The COVID-19 pandemic also played a prominent role in setbacks. During 2022, nationally, more than 320 human fatalities were reported, compared to less than 250 in 2021. In response, some legislative members called for an investigation of the failure of the national rabies prevention and control program to reach intended targets to eliminate human rabies by 2020 and the unmet declaration of the country to be rabies-free by 2022 (Cruz 2022). In lieu of canine rabies elimination throughout the archipelago by MDV, in addition to appropriate PEP, other strategies to reduce human deaths may include focused PrEP considerations for at-risk groups, such as pediatric populations (Quiambao et al. 2020, 2022).

The documented progress throughout the Americas and the focused attention via the BMGF projects helped spur additional initiatives. During 2015, a Pan-African Rabies Control Network (PARACON) meeting was held in South Africa to provide tools and coordination toward the elimination of canine-mediated human rabies deaths throughout the continent (Scott et al. 2015). Concomitantly with the PARACON outcome and based upon evidence acquired largely outside the region, the leadership of the WHO, WOAH, FAO, and other partners targeted an ambitious goal of global elimination of human rabies mediated *via* dogs (GEHRD) by 2030, focusing on LMICs in Africa and Asia (Anonymous 2015). Human prophylaxis, MDV, public education, and community engagement were considered crucial elements to attaining this 'Zero by 30' (ZBT) goal (WHO Rabies Modeling Consortium., 2020).

By the 21 century, epidemiological evidence showed that canine-mediated rabies was eliminated from all highly developed countries and increasingly controlled in middle-income countries through large-scale MDV campaigns and modern prophylaxis. To be clear, the GEHRD could be accomplished without actual canine rabies elimination by minimizing animal bites, preventing human rabies after exposure by PEP, and enhancing responsible dog ownership, but the risk of transmission of RABV from free-ranging dogs would remain (Mindekem et al. 2017). Such a first step is admirable and necessary but not economically sustainable. The long-term ultimate goal is complete elimination of canine rabies. Otherwise, the risk of human rabies acquisition from infected dogs remains, as seen in multiple situations.

Despite the wide availability of basic tools for such prevention and control, most LMICs, particularly throughout sub-Saharan Africa, still grapple with the first steps of the GEHRD (Zinsstag et al. 2017; Mbilo et al. 2021). Sub-Saharan Africa is second only to Asia in terms of the estimated global burden (Knobel et al. 2005; Hampson et al. 2015). After the announcement of the ZBT goal during 2015, few countries made substantive progress (Kanda et al. 2022). The reasons are multiple and share similarities with other NTDs. For example, in Chad, major barriers included economic barriers (e.g., vaccine costs, expense in transporting dogs, etc.), sociocultural (e.g., perceptions of the vaccine tainting dog meat for consumption, religious prohibitions on handling dogs, etc.) and institutional (unavailability of biologics, absence of training, etc.) aspects (Mbaipago et al. 2022). Such transdisciplinary concerns are relevant throughout many LMICs, as discussed in detail (Rupprecht and Salahuddin 2019; Tantawichien and Rupprecht 2020; Swedberg et al. 2022).

Since the 2015 GEHRD goal and ensuing COVID-19 pandemic, within tropical Old World hot spots, no country has eliminated canine-mediated human rabies deaths (Rupprecht et al. 2022). National surveillance databases and routine reporting mechanisms through PAHO/WHO and WOAH channels, together with regional disease conferences, confirm this reality. As one other aid to gauge progress, the Stepwise Approach Toward Rabies Elimination (SARE) uses monitoring tools for data-driven evaluation of strengths and weaknesses of rabies prevention and control programmes (Coetzer et al. 2016). With SARE scores between zero (for canine rabies endemic countries with no control) and five (signifying freedoms from canine rabies), current evidence indicates that canine-mediated rabies remains uncontrolled in much of Asia and Africa, with scores unavailable or between zero and one (Global Alliance and for Rabies Control (GARC) 2022). Optimistically, during 2018–2019, a few countries (e.g., Bhutan, the Philippines, Thailand, Vietnam, etc.) achieved SARE scores of ~ 3.5, approaching the ZBT goal (Global Alliance and for Rabies Control (GARC) 2022). Such progress resulted from long-standing free access to human PEP and dog vaccination (Tenzin 2012; Valenzuela et al. 2017; Anothaisintawee et al. 2019; Nguyen et al. 2019). Unfortunately, postpandemic COVID-19 disruptions in One Health implementation, with the detection of new rabies cases among dogs and humans, created setbacks throughout the region (Acharya et al. 2021; Lhendup and Dorji 2021; Ghai and Hemachudha 2022; Pham-Thanh et al. 2022).

While there have been significant attempts to manage canine-mediated human rabies in some parts of southern Asia through investment in millions of dollars by local authorities and international organizations, most countries still report human rabies deaths. For example, in Afghanistan, between 2017 and 2019, 36,959 animal rabies cases were recorded, with an estimated human rabies incidence of 5.7 per 100,000 population (Abdul-Jalil 2020). A national rabies prevention and control plan was developed but remains unimplemented. There are large numbers of free roaming dogs in the country and erratic dog vaccinations (Acharya et al. 2021). To date, Afghanistan has not undertaken a SARE assessment (Global Alliance and for Rabies Control (GARC) 2022).

China, second only to India with regard to the burden of human rabies, has decreased cases in most urban centers over the past decade. (Yue et al. 2022). Of 39 notifiable diseases, rabies ranked 3^rd^ in 2015. The country had a SARE score of 1.5 during 2019 (Global Alliance and for Rabies Control (GARC) 2022). Chongqing, China, is the world's largest municipality, with a population of ~ 32 million and an estimated ~ 1.3 million dogs, of which 200,000 are considered free roaming (Mission Rabies (MR) 2023). Between 2007 and 2016, 809 human rabies deaths were recorded (Qi et al. 2018). From information on 548 human cases, only 88% sought medical care, and none received PEP. Laboratory confirmation was only possible for a few of the offending dogs. During 2008, local authorities enhanced surveillance for dog rabies, improved access to PEP, and made canine vaccination compulsory. These measures reduced human deaths to an incidence of ~ 0.1/100,000 by 2013 and improved overall data quality (Qi et al. 2018; Mission Rabies (MR) 2023). Elsewhere in a bordering province, 59 human rabies deaths were recorded in Hunan during 2022 due to failures in access to proper wound care, RIG, and vaccine after exposure (Yang et al. 2022). Between 2010 and 2020, Shandong Province recorded 414 canine-mediated human rabies cases, with the majority in farmers (Zhang et al. 2022). Hence, while China has shown progress in reducing human fatalities in some large population centers, such as Beijing, PEP is not free for exposed persons, and health disparities exist in many rural areas.

At one time, Sri Lanka seemed poised to meet the ZBT. The country demonstrated support for multiple One Health applications, including enhanced surveillance in humans, domestic animals and wildlife; the feasibility of MDV and oral vaccination of free-ranging dogs; educational outreach; and support for human PEP (Perera et al. 2000; Perera et al. 2007; Matibag et al. 2009; Matsumoto et al. 2011; Matsumoto et al. 2013; Häsler et al. 2014; Karunanayake et al. 2014; Kanda et al. 2015; Harischandra et al. 2016; Kularatne et al. 2016; De Silva et al. 2017; Gamalathge et al. 2019). Moreover, thanks to the NGO Mission Rabies (MR), in collaboration with the local authorities, Sri Lanka vaccinated millions of dogs and sterilized at least 40,000 individual dogs (Sánchez-Soriano et al. 2019; Mission Rabies (MR) 2023). These efforts have resulted in a decline in human deaths from hundreds in the 1970s to 31 in 2021. However, the impacts of the COVID-19 pandemic, an unprecedented financial crisis in 2021, and major sociopolitical strife have impacted the public health budgetary allocation for rabies, leading to a shortage in PEP for the foreseeable future (Kanda et al. 2021). At least 28 people succumbed to rabies last year.

In Vietnam, based on surveys from 2020 to 2021, rabies was the most serious of five priority zoonoses (Pham-Thanh et al. 2022). Between January and August 2022, at least 40 human rabies deaths mediated *via* dogs were recorded. This was an increase compared to the previous years (NewsDesk 2022). The department of preventive medicine recently recommended vaccinations for dogs and cats, confinement of dogs, and basic preventative measures, such as washing bite wounds with soap and water and seeking care in the event of exposure. Regardless, 20 provinces witnessed increased human rabies deaths in recent years.

In Bangladesh, rabies is endemic and accounts for 2,000–2,500 human deaths annually (Mondal and Yamage 2014; South Asian Association for Regional Cooperation (SAARC), 2019). The country has recently prioritized rabies, with a SARE score of 2.5 in 2019 (Global Alliance and for Rabies Control (GARC) 2022). A coordinated multisectoral One Health effort, with the support of international partners and a substantial investment of $33 million in 2017–2022 across 65 district municipalities, resulted in a decline in annual rabies incidence (Li et al. 2019). The country also trained dog catchers and vaccinators in 2019 to support MDV (SAARC 2019). However, the presence of many free-roaming dogs, poor access to PEP, and limited surveillance capacity hamper efforts to attain the ZBT target.

In contrast to the few examples in Asia, no African countries achieved a SARE score above 2.5 (Global Alliance and for Rabies Control (GARC) 2022). One factor to claim true freedom from rabies is an effective laboratory-based surveillance system to detect both human and animal cases. However, the weak capacity for routine surveillance in most countries throughout Africa provides insufficient data to inform investment in rabies prevention and control (Mbilo et al. 2021; Rupprecht et al. 2022). Moreover, the COVID-19 pandemic refocused available funding, personnel, and diagnostics (Nadal et al. 2022a, b). Other major limitations include: a shortage of trained local champions; lack of an evidence-based national strategic plan to prioritize scarce resources; limited One Health prioritization; and poor healthcare systems, that do not allow for prompt interventions in the event of RABV exposure (Mshelbwala et al. 2021). Moreover, such health care limitations lead many dog-bite victims to resort to traditional healers involving the use of herbs, potions and other concoctions (Mshelbwala and Weese 2017; Audu et al. 2019; Ghosh et al. 2020; Rana et al. 2020).

While some national governments in Africa have been supported in developing a national strategic plan to gain political support locally, implementing such a plan has been a major challenge. Unlike in Latin America, many efforts are driven by NGOs and investigators from Western countries working in prior European colonies, rather than any internal bootstrap approach, without the prospect of any long-term funding from external investors. For example, in collaboration with FAO, the Global Alliance for Rabies Control (GARC) delivered capacity-building workshops and assessments and workshops across Africa. In addition, GARC complimented the long-term MDV effort by vaccinating over 37,000 dogs in the northern region of Malawi in 2022 and supported Zanzibar, an island that has made significant progress in enhancing surveillance toward ZBT, with a SARE score of 2.5 during 2018 (Global Alliance and for Rabies Control (GARC) 2022).

During 2022, the NGO Mission Rabies (MR) reached a global milestone of vaccinating over two million dogs against rabies since its inception in 2013 (Mission Rabies 2023). In addition, MR supported community education and enhanced surveillance in multiple hot spot locations, including Cambodia, Ghana, India, Malawi, Mozambique, Sri Lanka, Thailand, Uganda and Zambia, delivering over 200,000 vaccines annually to dogs. The success of MR interventions has been due in part to the recruitment of international and local volunteers. Unlike the experience of MR, which helps deliver MDV using tools that allow monitoring of teams, personnel, supplies and impact, most interventions rolled out by local authorities in Africa remain short term and uncoordinated, without proper data documentation to measure impact.

In response to such regional needs, the FAO has been training veterinarians and para-veterinary professionals under an In-Service Applied Veterinary Epidemiology Training (ISAVET) program (Fasina et al. 2021). This program aims to strengthen local and para veterinarians' capacity through earlier detection and response to outbreaks in a timely manner through access to modern epidemiological tools. This can alleviate the current drawbacks in rabies management for Africa. There is an overdue need to actively involve private veterinarians and academics in planning and implementing MDV and recruiting students as volunteers, similar to the model of MR. University laboratories could also be better utilized for improved rabies surveillance. Thus, in principle, the GEHRD is feasible, with long-term investments in canine rabies surveillance, large-scale, data-driven MDV, public awareness creation, and application of WHO recommendations on human PEP (Cleaveland and Hampson 2017; Wallace et al. 2020).

For example, a recent MR project in the state of Goa, India, reported the elimination of human rabies and a greater than 90% reduction in monthly canine rabies (Gibson et al. 2022). Three predictable items formed the pillar of this program: MDV, targeting owned, confined, and free-roaming dogs, using a GIS-aided mobile phone app, guiding vaccination teams via assigned polygonal areas; the delivery of rabies education to school children and teachers on basic related knowledge, including how to avoid dog bites and what to do if bitten by a dog to receive medical care such as PEP; and enhanced surveillance through real-time reporting, using dedicated hotlines centrally managed to request dog vaccination and report unhealthy dogs. Based on reports, a full-scale investigation commenced to assess risk, including diagnosis, using the DFAT, rapid test kits for screening, and RABV sequencing to explore the utility of applied molecular epidemiology in disease forecasting management (Gibson et al. 2022). Plans are underway to repeat the Goa experience in other locales throughout India (Fig. 8).

Given such recent local success on the subcontinent, how would other national authorities with more limited resources be convinced to prioritize such a venture? An evidence-based evaluation of the national rabies burden (canine and human rabies) is critical as a prerequisite to driving such government investment. This would also include a plan to estimate dog populations, acquire adequate vaccines, supplies and staff, develop a program to vaccinate at least 70% dogs, and enhance laboratory capacity for canine rabies surveillance to assess success, including geographical risk assessments to better guide evidence-based interventions and public awareness creation to break the transmission cycle, consistent with long-standing recommendations (Lembo et al. 2012). As one example from West Africa, recent investigations in Nigeria began in an effort to provide multiple lines of evidence to contribute to more informed investment in national rabies prevention and control toward ZBT (Mshelbwala et al. 2021, 2022). Similar efforts are crucial elsewhere in Africa to drive evidence-based interventions toward the ZBT target.

**Fig. 7:**
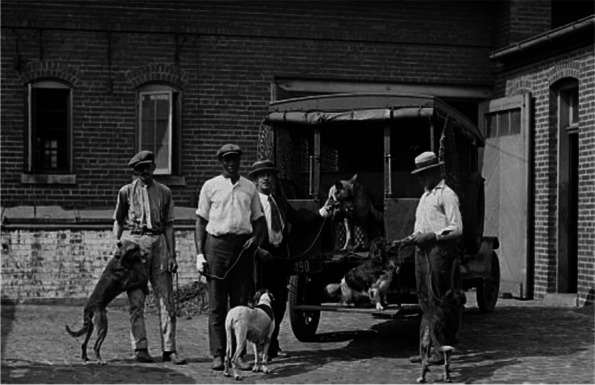
Dog catchers, 1924. Library of Congress, Prints and Photographs Division, Washington, D.C. 20,540 USA http://hdl.loc.gov/loc.pnp/pp.print

**Fig. 8:**
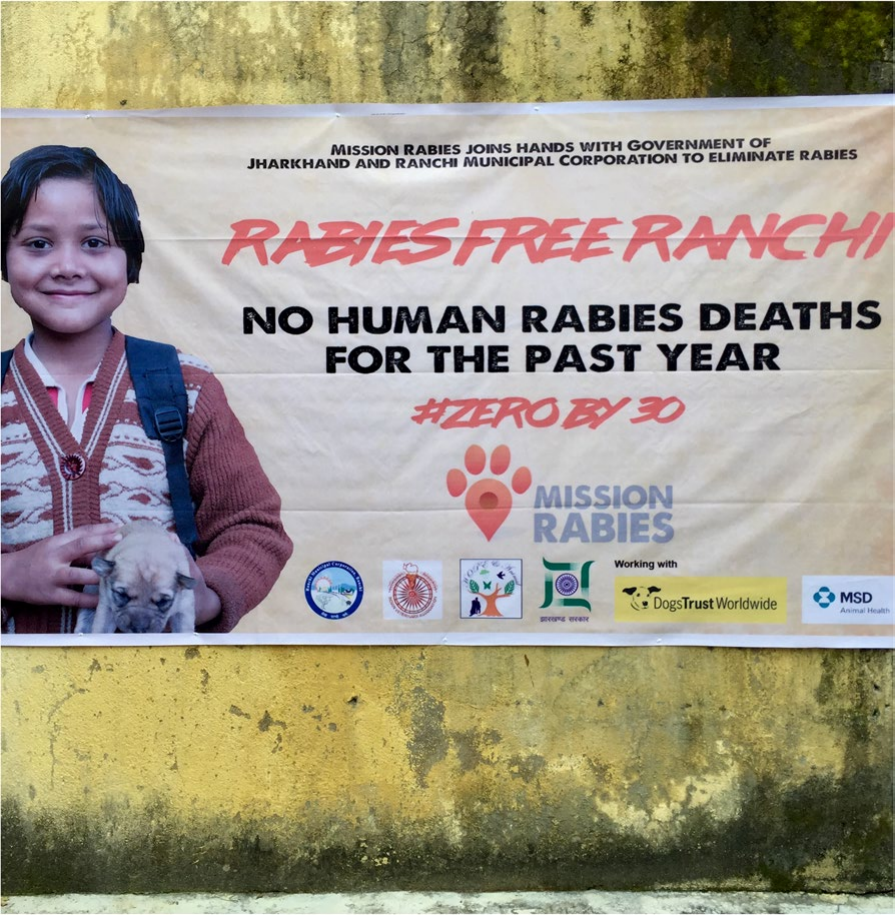
Street poster, emulating responsible dog ownership, downtown Ranchi, India

However, with the approaching deadline of 2030 and with the evidence at hand, the ZBT target does not appear entirely feasible. Dogs thrive wherever there are humans, from the Arctic to the tropics. Livestock predation, bites and infectious diseases such as rabies are just a few of the recognized conflicts. Historically, dog ‘whippers’ or ‘wardens’ were employed to harm, catch, and kill dogs (Figs. 5 and 6). In Western countries, concomitant with the era of vaccination in the early twentieth century, with an increasing concern for general welfare, free-ranging animal management consisted largely of local removal, with captured individuals held in shelters for adoption or euthanasia (Fig. 7). Parallel industries in LMICs are much more limited in scope and operation, but gradual improvements in animal welfare have begun. Local education campaigns toward the promotion of vaccination, contraception and more holistic practices have had a major impact in some parts of Africa and Asia (Fig. 8). However, animal birth control strategies that spay/neuter and vaccinate dogs, returning them to the streets, do not alleviate all societal concerns. In turn, residents may demand the removal of dogs from their neighborhoods or result in the recidivism of local culling. Without further appreciation of such ignored facets as the local socioeconomic dynamics for broader support of sustainable animal management and responsible pet ownership, together with relevant operational research on the community ecology of dogs in line with vaccination, the likely success of the ZBT ‘…*is slight and hardly realistic*…’ (Subedi et al. 2022).

## Riposte for therapy of clinical cases

In contrast to reasonably clear disease prevention and control considerations, one of the major, unresolved ‘holy grail’ issues in the field concerns the actual treatment of clinical rabies. Ever since the recognition of Matthew Winkler as the first documented human recovery during the 1970s, the hope continued in the quest for other survivors, but without a clear path or certainty of protocol, beyond excellent clinical support (Hattwick et al. 1972). Notably, there are many hurdles in considering such human treatment (Table 2). Clearly, pathobiology remains a fertile area, as many basic ‘infection and immunity’ features, such as cell signaling pathways (especially innate immune response), including the upregulation of TLRs and chemokine receptors for the recruitment of leukocytes and cytokines in the CNS, among others, remain an enigma.

**Table 2:** Challenges in the implementation of human rabies treatment

Number	Challenges
1	Historical fear and ignorance of a virulent pathogen
2	Extensive CNS involvement by time of diagnostic suspicion
3	Not an outpatient option
4	Unvaccinated healthcare workers
5	Need for a ‘dream team’ of intensive care specialists
6	Dedicated intensive care facility
7	No licensed antiviral drugs, requiring heroic use
8	Uncertain pathobiological targets and mechanisms
9	Delivery of biologics across the BBB
10	Clinical and diagnostic difficulties in monitoring patient status
11	Costs of therapy
12	Extensive rehabilitation
13	Quality of life in the face of neurological deficits
14	Religious, cultural, and ethical concerns on ‘experimentation’
15	High probability of failure

Recognizing such formidable drawbacks and that previous survivors had received PEP before the onset of illness, recommendations were made for aggressive considerations to management, if attempted, and the imperative need for research advances in pathobiological understanding and improvement in available tools (Jackson et al. 2003). During 2004, Jeanna Giese, a 15-year-old from Wisconsin, USA, was exposed to a bat but did not receive PEP. Approximately 1 month later, Jeanna developed signs compatible with rabies. She was hospitalized and treated with therapeutic coma and N-methyl-d-aspartate receptor antagonist therapy, becoming the first documented survivor without a history of vaccination (Willoughby et al. 2005). Afterwards, adaptations occurred of this initial regimen, termed the Milwaukee Protocol (MP). Recognizing a relationship between a favorable clinical outcome and the induction of RABV antibodies in the CSF (such as in Jeanna’s situation), critical care suggestions were made to especially target rabies patient selection for treatment toward those with early VNA onset (Wilde et al. 2008). Multiple additional treatments with MP were attempted, with many failures, while Kaplan–Meier survival curves after initiation appeared significantly longer compared to contemporary controls (Medical College of Wisconsin 2022). Controversy over the MP persists (Jackson 2018). Nevertheless, Jeanna’s dramatic recovery provoked a veritable renaissance on the topic that rabies is not invariably fatal. This reinvigorated a drive for new evidence-based tools for administration during attempted treatment (not just via in vitro models), such as effective antiviral drugs and the delivery of efficacious antibodies across the blood–brain barrier (Du Pont et al. 2019; de Melo et al. 2022; Kimitsuki et al. 2022). Another method may be in situ production using recombinant viruses (Huang et al. 2021). For example, after IV administration, RABV antibodies are expressed within the brains of animals within days following adeno-associated viral vector antibody gene therapy (Baker et al. 2022).

As opposed to the obvious drawbacks of ad hoc nature human cases and experimental treatment, is there an alternative to other species in the interim? For example, given the prevalence of naturally occurring rabies in dogs, adoption of a treatment protocol would not be wanting for a shortage of cases or restrictions due to locale. In addition, there is a built-in welfare component to ensure that such animals are removed as a public health measure from the streets, where they are otherwise considered pariahs and killed (not euthanized) outright. Veterinarians are much more likely to receive PrEP in advance, rather than as an afterthought, due to occupational exposures and have a professional familiarity with zoonoses such as rabies. Moreover, the comparative costs (i.e., facility, equipment, drugs, staff, etc.) of operation should be considerably less expensive than in human medicine. As such, a canine model offers multiple advances to understanding the basic pathobiological mechanisms in a natural reservoir that have thwarted progress in humans and is also relevant for modern medical interventions that have been otherwise difficult (e.g., intubation, long-term anesthesia, imaging, sample acquisition, etc.) in some smaller-bodied taxa (i.e., rodents) for practical application (Knobel et al. 2022).

## Robust biologics

Practical rabies management is not possible without the availability of biologics, as recognized for more than a century. In contrast to many emerging infectious diseases, vaccines for rabies are broadly pure, potent, safe and efficacious (Banyard et al. 2018). Rabies vaccines for domestic animals have a minimum duration of immunity claims of three years or longer (NASPHV 2016).

For humans receiving PrEP, no failures were reported over more than four decades of surveillance (Rao et al. 2022). In addition to the use of PrEP for individuals under occupational risk (e.g., veterinarians, laboratory workers, wildlife biologists, etc.) or travelers, administration to communities at risk is also being reevaluated (Royal et al. 2022; Taylor et al. 2022). Considering more than 12 million exposures per year, human PEP is also highly effective when administered appropriately and in a timely manner, with only 122 failures identified between 1980 and 2022 (Whitehouse et al. 2022). The institution of shorter schedules and dose-sparing strategies for both PrEP and PEP provides critical biologics to those most in need (World Health Organization (WHO), 2018). In addition to humans and domestic animals, oral vaccination of wildlife has led to widespread prevention and control of rabies in western Europe and North America. Oral vaccines, proven in concept and action for wildlife, may be perceived as a game changer if applied toward free-ranging community dogs (Bobe et al. 2023). However, this is not a new concept or a panacea and will only augment success where at least a rudimentary prevention and control program already exists, including MDV and laboratory-based surveillance (Cliquet et al. 2018). All of these applications were in effect during the twentieth century.

Additional technological applications have broadened the spectrum even further for prevention and control with regard to new biologics in use or under development (Ertl 2019). For example, after initial design in 1978, monoclonal antibodies (MAbs) were finally being used in human PEP as an alternative to RIG (de Melo et al. 2022). This was in no small way prompted by a major collaboration among the WHO Collaborating Centers for such technology transfer and realization postconcept (Müller et al. 2009; Phoolcharoen et al. 2019). Besides prophylaxis, MAbs are also under consideration for treatment (de Melo et al. 2020). Although the use of virus-like particles (VLPs) was considered for veterinary applications for several years, during 2022, VLP nanoparticles were used for a 1-week, 3-dose human application (Ravish et al. 2021). Most human vaccines are not adjuvanted, but a polyinosinic-polycytidylic acid-based adjuvant stabilized with kanamycin and calcium (acting as a TLR3 agonist) was well tolerated and immunogenic when tested in healthy volunteers (Wijaya et al. 2017). Highly purified, serum-free human vaccines are on the horizon (Pichon et al. 2022). Using a very different application via nucleic acid delivery, after two administered doses of a RABV glycoprotein mRNA encapsulated in lipid nanoparticles, all recipients developed VNA (Aldrich et al. 2021). To reduce the need for a return visit to the clinic for a booster dose and consider only a single dose of vaccine for PrEP, clinical trials have begun to evaluate a simian adeno-RABV product (Jenkin et al. 2022). Many of these new and existing biologics, whether administered to humans or animals, are intended for a single application of one dose per individual. In contrast, recently, the idea of ‘self-spreading’ vaccines has re-emerged into the foreground (Griffiths et al. 2023).

## A role for ‘self-spreading’ vaccines?

Vaccines represent one of the most successful biomedical technologies in history. In a One Health context, human rabies vaccines were created during the late nineteenth century, dog vaccines were applied a century ago, and wildlife vaccines were developed over 50 years ago. Paradoxically, regardless of the pathogen, such success brings a risk of failing to adequately appreciate all the diverse challenges that have been overcome in the vaccine field to date (Messinger Cayetano and Crandall 2020). One example is the concept of ‘self-spreading’ vaccines (Nuismer et al. 2016, 2018; Scudellari 2016; Bull et al. 2018; Bakker et al. 2019; Cogley 2020; Nuismer and Bull 2020). In contrast to all licensed vaccines to date that are delivered to the individual by parenteral or mucosal routes, self-spreading vaccines are replication competent and intended to spread autonomously in the environment between individuals, often with epidemic-like dynamics. This outwardly attractive idea would enable a small fraction of a target population to be inoculated, with an expectation of achieving a population-wide herd immunity effect, which may extend to as yet unborn members of the same population at the time of initiation.

The basic concept of self-spreading vaccines is not new and occurred to generations of virologists and vaccine developers (Browne 1991; Ross 1998; Angulo and Bárcena 2007; Angulo and Gilna 2008; Lentzos et al. 2022). For example, the tools necessary to assemble such genetically modified biologics were adequate in the 1990s to generate a self-spreading vaccine aimed at protecting wild native Spanish rabbits from myxomatosis and rabbit hemorrhagic disease (Bárcena et al. 2000; Torres et al. 2001; Angulo and Bárcena 2007). This product was tested in a field release on a Spanish island, although efforts to license the vaccine (under the name LAPINVAC) with the European Medical Agency were ultimately abandoned by 2007.

Self-spreading vaccines are genetically modified organisms, typically tested in biosafety level 3 or 4 facilities (Bárcena et al. 2000; Tsuda et al. 2011). Somewhat confusingly, self-spreading vaccines exist under a multitude of equivalent terms that may include transmissible, contagious, horizontally transferable, self-disseminating, or founder-based. A hypothetical term, “transferable vaccine”, has also been introduced to denote self-spreading vaccines, where intended transmission only occurs to those in direct contact with the originally inoculated individuals (Nuismer and Bull 2020; Technology Networks 2022). However, we are unaware of any evidence that such a class of licensed agents exists, particularly as viral transmissibility is a dynamic parameter in complex environmental situations.

Currently, there are several proposals for self-spreading vaccines:

- 1. Vaccination of African nonhuman primates to inhibit their infection by Ebola virus, with the aim of limiting their capacity to act as a wildlife reservoir for transmission to humans (PREEMPT 2018, 2022; TVG 2021).
- 2. Vaccination of rodent species in West Africa to inhibit their infection by Lassa fever virus, with the aim of limiting their capacity to act as a wildlife reservoir for transmission to humans (PREEMPT 2018, 2022; Regulatory News Service 2022).
- 3. Vaccination of North American bat species to reduce their susceptibility to an emergent fungal infection for the purposes of bat conservation against White Nose Syndrome (Rocke et al. 2019; USDA-APHIS 2019).
- 4. Vaccination of vampire bats is currently restricted to Central and South America to inhibit their infection by RABV, with the aim of limiting their capacity to act as a wildlife reservoir for transmission to humans and other animals (Bakker et al. 2019; Streicker et al. 2022).

In this communication concentrated on rabies, we focus on the fourth proposal, involving vampire bats (although such a concept could be applicable to other reservoirs). Vampire bats are distributed throughout Central and South America, from Mexico to Argentina (Johnson et al. 2014). As obligate parasites of vertebrates, RABV transmission occurs during feeding by these hematophagous species. Ideally, population reduction is used proactively to manage vampire populations. In bite victims, rabies prevention consists of individual vaccination of humans and domestic animals. Even allowing for the undoubted unevenness of regional disease monitoring and control efforts, the incidence of human rabies has declined to the point that the vast majority of cases now occur outside of the Americas (WHO 2023). Due unambiguously to the collective success of MDV and PEP efforts, most of the residual ‘vectoring’ of rabies in Central and South America is now due to vampire bats. This success using traditional biologics has contributed to the proposal to use self-spreading vaccines to limit the capacity of vampire bats to transmit RABV, in part by exploiting their roosting and grooming behaviors to facilitate viral vaccine spreading (Bakker et al. 2019; Nuismer and Bull 2020).

Recent modeling has suggested the intuitive finding that self-spreading vaccines are more resource efficient than conventionally deployed biologics (Nuismer et al. 2016, 2018, 2019). The question of whether self-spreading viral vaccines are likely to be operationally useful in their target remains largely unaddressed. For example, will they simultaneously be ‘transmissible enough’ to be successful and still be perceived as ‘sufficiently controllable’ by regulators and generally acceptable to the public? Given the variability in continuously dynamic model parameters, such as viral transmissibility, and the stability of genetic modification(s) introduced to the vaccine virus genome, it is difficult to discern the extent to which human communities could be reasonably encouraged to rely on such management interventions for infectious spillover protection. For example, such applications would be contrasted otherwise to well-resourced and coordinated application of available and conventionally deployed public health and veterinary technologies to date. As such, the literature promoting self-spreading vaccines in the context of zoonotic disease control frequently focuses on the exact goal of local elimination of pathogens from wildlife, rather than their stable suppression, per se. Rather lamentably, there is a conspicuous and repeated effort to rebrand current One Health methods as somehow “reactive”, whereas self-spreading vaccines are presented as innovatively “proactive” (Kerlin 2019). Such trivialization of the achievements of successive generations of researchers and local community control practitioners could be viewed as falling squarely within the paradox of success (Messinger Cayetano and Crandall 2020).

During the 1980s to 2000s, other programs considering self-spreading vaccines collapsed (Lentzos et al. 2022; Reeves 2022). Regardless of whether current proposals progress to the point that they could be argued to be not only safe but also trustworthy, useful, and effective, it is self-evident that in the current media environment, such concepts for self-spreading vaccines may construe a significant misinformation threat. This should be viewed in light of the recent erosion of confidence and vaccine hesitancy in some, but not all, parts of the globe (de Figueiredo et al. 2020). Given the importance of public trust to not only vaccine deployment but also the strategically essential capacity to innovate and develop new conventionally deployed biologics, caution by both proposers and funders of self-spreading speculations would appear to be warranted. In the absence of the need for technological breakthroughs, the speed at which self-spreading vaccines could be developed is quite short. Over the past three years, self-spreading vaccines for Lassa fever virus in rodents and Ebola virus in primates have been developed in Germany, the USA, and the UK (PREEMPT 2022; Regulatory News Service 2022; Technology Networks 2022). Furthermore, the first open field trial of the rabbit vaccine during 1999 predated any scientific publications on self-spreading vaccines (Torres et al. 2001). Such precedence appears problematic in the absence of international notifications for cross-border export of self-spreading vaccines or experimental field releases of veterinary self-spreading vaccines. As the broader vaccine community focuses on moving away from higher risk approaches where effective alternatives can be developed (i.e., replacing live attenuated vaccines generated by selection), there is considerable potential for the whole field to be represented by a small group of funders and investigators (mostly evolutionary biologists), moving rapidly in the direction of increasing not only the risk profile of vaccines but also the potential for misunderstanding or misinformation (transmissiblevaccines.org 2022). In the context of rabies prevention and control, it is reasonable to ask to whom self-spreading vaccine proposals would appear attractive, particularly if licensed biologics were being promoted and used more widely in at-risk populations. For example, how would success be measured? In the case of rabies, would exposed individuals still receive PEP? What techniques could manage vampire bat populations and their obligatory feeding behavior upon humans, domestic animals, and wildlife? At the very least, self-spreading RABV vaccines are no magic bullet, and such proposals involve multiple ethical, regulatory, scientific, and social issues, in need of much broader discussion.

## Conclusions

Recognizing the 200^th^ anniversary of his birth, aficionados of Pasteur would likely agree that ‘chance favors the prepared mind’, as applied to the considerable contributions that flowed thereafter, following his celebratory accomplishments on rabies vaccination (Tarantola 2017). When compared to the past century, major scientific and operational advances occurred in nearly all aspects of the field, providing a major example of One Health in action (Table 3). By comparison, given the underlying technical prerequisites needed for laboratory-based surveillance, vaccine-driven herd immunity, and basic epidemiological understanding that was central to the true eradication of smallpox and rinderpest, one might understand the tendency to view such significant zoonoses as rabies in the same light. Unfortunately, these are different realities. Smallpox was highly communicable, restricted to one host, killed millions annually and was eradicated globally by affordable vaccination for the public good under the auspices of the WHO, with ongoing issues related to other orthopoxviruses today (Gieryńska et al. 2023). In contrast, although rinderpest was not zoonotic and involved more than a single reservoir, its economic consequences upon ruminants (and the farming communities dependent upon them) were devastating to LMICs, prompting its eradication via vaccination (producing life-long immunity) under the auspices of the FAO (Jeggo and Roeder 2021). By simple comparison to these only two eradicated diseases to date (and similarly with polio, now more than two decades long overdue), such terminology is not applicable to rabies, promising much more than could ever be achieved.

**Table 3:** Paradigm shifts in rabies detection, prevention, control & treatment in the 21^st^ century

Topic	Change	Reference
Etiology	Classical rabies virus recognized as the most important member of the genus, but > 17 putative or recognized lyssaviruses, including highly divergent viral spp.	Fooks et al. 2021
Diagnosis	Since the early twentieth century, microscopic recognition of intracytoplasmic ‘viral factories’ within neurons, now supplemented by highly sensitive, specific, and economical tests for rapid ante- and postmortem laboratory confirmation and enhanced point-of-care field surveillance development	World Health Organization (WHO), 2018
Pathobiology	In addition to exposure via transdermal, mucosal and aerosol routes, transmission via solid organ and tissue transplantation	Srinivasan et al. 2005
Treatment	Survivors, without a history of prior vaccination	Willoughby et al. 2005
Safety	Abandonment of nerve tissue-based vaccines by safer alternatives	Gongal & Sampath 2019
Vaccine potency	In vitro alternates to the use of animal testing in the NIH test	Stokes et al. 2012
Passive immunity	Monoclonal antibodies used increasingly in humans	Sparrow et al. 2019
Improvement in biologics	Multiple novel biologics licensed or in human clinical trials	Ertl 2019
Prophylaxis	Efficacious and economical dose-sparing and 1-week strategies	WHO Rabies Modeling Consortium 2019
Wildlife rabies vaccination	Elimination of rabies in red foxes in western Europe, and Texas gray fox and coyote rabies virus variants in USA	Mähl et al. 2014; Maki et al. 2017; Vos et al. 1999
One Health	Global program for elimination of human rabies caused via dogs by 2030	Abela-Ridder et al. 2016
Advocacy	Inauguration of World Rabies Day	Centers for Disease Control Prevention (CDC), 2007

Whereas progress in rabies prevention and control is inarguable, eradication does not apply as a term in the objective light of considerable viral and host diversity and the subjective socioeconomic bias of neglect and disparity. In contrast to eradication, based upon the major regional success in the Americas, the GEHRD might seem achievable, conducted with twentieth century tools (although even here limitations are obvious and continuing). Additionally, the ZBT appeared more reasonable at its forecast during 2015, especially with the added promise of global collaboration and the prescient reliance upon the technical shape of things to come (Abela-Ridder et al. 2016). Compared to other diseases, rabies is a rather low bar to achieve reasonable prevention and control by conservative standards, overshadowed only by the even lower bar of political commitment and resources enacted to date. If two of the largest growing global economies (*i.e.*, China and India) would more actively advocate for and financially support rabies elimination campaigns among dogs in ASEAN/Far East and the Indian subcontinent, more than 50% human rabies deaths would be prevented.

The COVID-19 pandemic refocused time, talent, and resources from all NTD. Progress in rabies surveillance, prevention, and control has suffered, especially in LMICs. In retrospect, PAHO set at least four different target dates for elimination in the Americas over the past 30 years, but the region is still not free of canine rabies (Del Rio Vilas et al. 2017). As such, in light of such major setbacks and the minimal SARE scores representative today as one bellwether in most canine rabies enzootic countries, a global reappraisal is warranted unless substantive global support materializes (such as from GAVI), together with a prioritized political will for the application of modern human PEP and appropriate MDV (Thumbi et al. 2022). Before elimination can accrue tomorrow, practical control needs to begin today with available tools and techniques.

## Data Availability

Not applicable.

## Abbreviations

BMGF: Bill & Melinda Gates Foundation
CDC: Centers for Disease Control & Prevention
CST: Cross species transmission
DFAT: Direct fluorescent antibody test
FAO: Food and Agriculture Organization of the United Nations
GARC: Global Alliance for Rabies Control
GAVI: The vaccines alliance
GEHRD: Global elimination of human rabies from dogs
ICTV: International Committee for the Taxonomy of Viruses
ISAVET: In-Service Applied Veterinary Epidemiology Training
KZN: KwaZulu-Natal
LMIC: Lower- and middle-income countries
MAb: Monoclonal antibodies
MDV: Mass dog vaccination
MR: Mission Rabies
NASPHV: National Association of State Public Health Veterinarians
NTD: Neglected Tropical Diseases
OIE/WOAH: World Organization for Animal Health
PAHO: Pan American Health Organization
PARACON: Pan-African Rabies Control Network
PEP: Postexposure prophylaxis
PrEP: Preexposure prophylaxis
RABV: Rabies virus
RIG: Rabies immune globulin
RFFIT: Rapid fluorescent antibody test
RTSL: Resolve To Save Lives
SAARC: South Asian Association for Regional Cooperation
SARE: Stepwise approach to rabies elimination
VLP: Virus-like particle
VNA: Virus neutralizing antibody
WHO: World Health Organization
WRD: World Rabies Day
ZBT: ‘Zero by thirty
